# Impact of body composition metrics extracted from QCT and the corresponding nomogram on the evaluation of survival prognosis in AML patients

**DOI:** 10.3389/fonc.2025.1498024

**Published:** 2025-09-09

**Authors:** Tao Yuan, Yaxin Zhang, Yawu Liu, Guodong Gao, Guanmin Quan

**Affiliations:** ^1^ Department of Medical Imaging, The Second Hospital of Hebei Medical University, Shijiazhuang, China; ^2^ Department of Clinical Radiology, Kuopio University Hospital, Kuopio, Finland; ^3^ Department of Neurology, University of Eastern Finland, Kuopio, Finland

**Keywords:** acute myeloid leukemia, prognosis, physical composition, quantitative computed tomography, nomogram

## Abstract

**Objectives:**

This study aimed to investigate whether the body composition metrics extracted from quantitative CT (QCT) are associated with the survival prognosis of acute myeloid leukemia (AML) patients and to evaluate the impact of a nomogram based on QCT and clinical–physical factors in predicting the prognosis of AML.

**Methods:**

The clinical factors and QCT metrics of 127 AML patients undergoing initial chest CT were analyzed retrospectively. The AML patients were divided into favorable and poor prognosis groups based on the threshold of median overall survival (OS). A QCT metrics- and clinical factors-derived nomogram was constructed using multivariate Cox regression. The performance of the nomogram was assessed with a receiver operating characteristic curve (ROC), calibration curve, and decision curve analysis (DCA).

**Results:**

Compared to patients in the favorable survival prognosis group, patients with poor prognosis were older (*p* = 0.027), had higher risk stratification (*p* = 0.006), more positive minimal residual disease (MRD) (*p* = 0.014), lower skeletal muscle index (SMI) (*p* = 0.045), and a higher incidence of volumetric bone mineral density (vBMD) ≤ 120 (*p* = 0.035). Older age, higher risk stratification, positive MRD, and SMI < 15.74cm^2^/m^2^ were independent risk factors for poor prognosis in AML patients. The areas under the ROC curve (AUCs) of the nomogram, which included SMI and independent clinical factors, for predicting 1- and 2-year OS were 0.792 and 0.794, respectively. The calibration curve and DCA demonstrated the good performance of the nomogram prediction model.

**Conclusions:**

Sarcopenia revealed by QCT, integrated into a nomogram with age, risk stratification, and MRD, can facilitate individualized prediction of survival prognosis in AML patients.

## Introduction

Acute myeloid leukemia (AML) is the most common subtype of acute leukemia in adults, with an incidence of 4.3 per 100,000 per year. The prognosis of most AML patients is poor, with a 5-year survival rate of approximately 24% (1). The poor prognosis of AML has been ascribed to several factors, including molecular genetic abnormalities at initial diagnosis, treatment regimen, and age. In recent years, it has been found that body composition factors are also associated with the prognosis of AML. Previous studies showed that sarcopenia was also associated with a poor prognosis in AML. However, the association of adipose tissue content with the prognosis of AML has yet to be explored (2, 3).

Currently, the body composition can be evaluated with clinical factors and quantitative imaging analysis. Clinical evaluation methods include body mass index (BMI) and bioelectrical impedance analysis. Underweight status, which is defined by lower BMI, has been found to be associated with poor outcomes in AML patients (4). However, there is controversy about the relationship between adipose tissue and the prognosis of AML. Sarcopenia, which was diagnosed based on skeletal muscle mass extracted by bioelectrical impedance analysis, was an independent factor for shorter overall survival (OS) in patients with AML (5). Unfortunately, BMI does not directly reflect adipose tissue distribution and muscle mass in AML patients. Despite its simplicity and convenience, the accuracy and specificity of bioelectrical impedance analysis are lower due to the influence of hydration status, food intake, and physiological movement on the measurement (6). Imaging techniques for evaluating body composition mainly include dual-energy X-ray absorptiometry (DXA), magnetic resonance imaging (MRI), and computed tomography (CT). DXA is mainly used in the evaluation of bone mineral density and can also quantify the content of a single body part and adipose tissue content. The accuracy of body composition measurement with DXA is susceptible to the influence of surrounding lesions, such as osteophytes and vascular calcification. In clinical practice, MRI is limited in the evaluation of body composition due to its disadvantages, including expense, time consumption, and low spatial resolution. Notably, quantitative CT (QCT) is a promising imaging method for the assessment of body composition because of its precise anatomic localization, ability to distinguish different structures, and direct evaluation of muscle, adipose tissue, and bone (7). It has been proven that the area of abdominal muscle and adipose tissue and the bone mineral density of the lumbar spine obtained by QCT were correlated with the corresponding metrics of the whole body. Thus, the measurement of abdominal structures could reflect the body composition of the whole body (8). Recently, the preliminary association analysis between sarcopenia and prognosis has also been highlighted in AML. Nevertheless, the association between quantitative adipose tissue metrics and survival prognosis has yet to be determined due to insufficient studies and their inconsistent conclusions (2, 3).

Therefore, the purposes of this study were to explore the prognostic impact of QCT metrics on survival prognosis evaluation and to determine whether the nomogram, based on the combination of clinical variables and QCT metrics, has better performance in assessing survival outcome in AML patients.

## Methods

### Study participants and data collection

From May 2020 to May 2021, consecutive patients who were newly diagnosed with AML were included. All these patients had QCT data extracted from routine chest CT examinations on admission. This study was approved by the review board of The Second Hospital of Hebei Medical University. Written informed consent was waived due to its retrospective nature.

**Figure 1:**
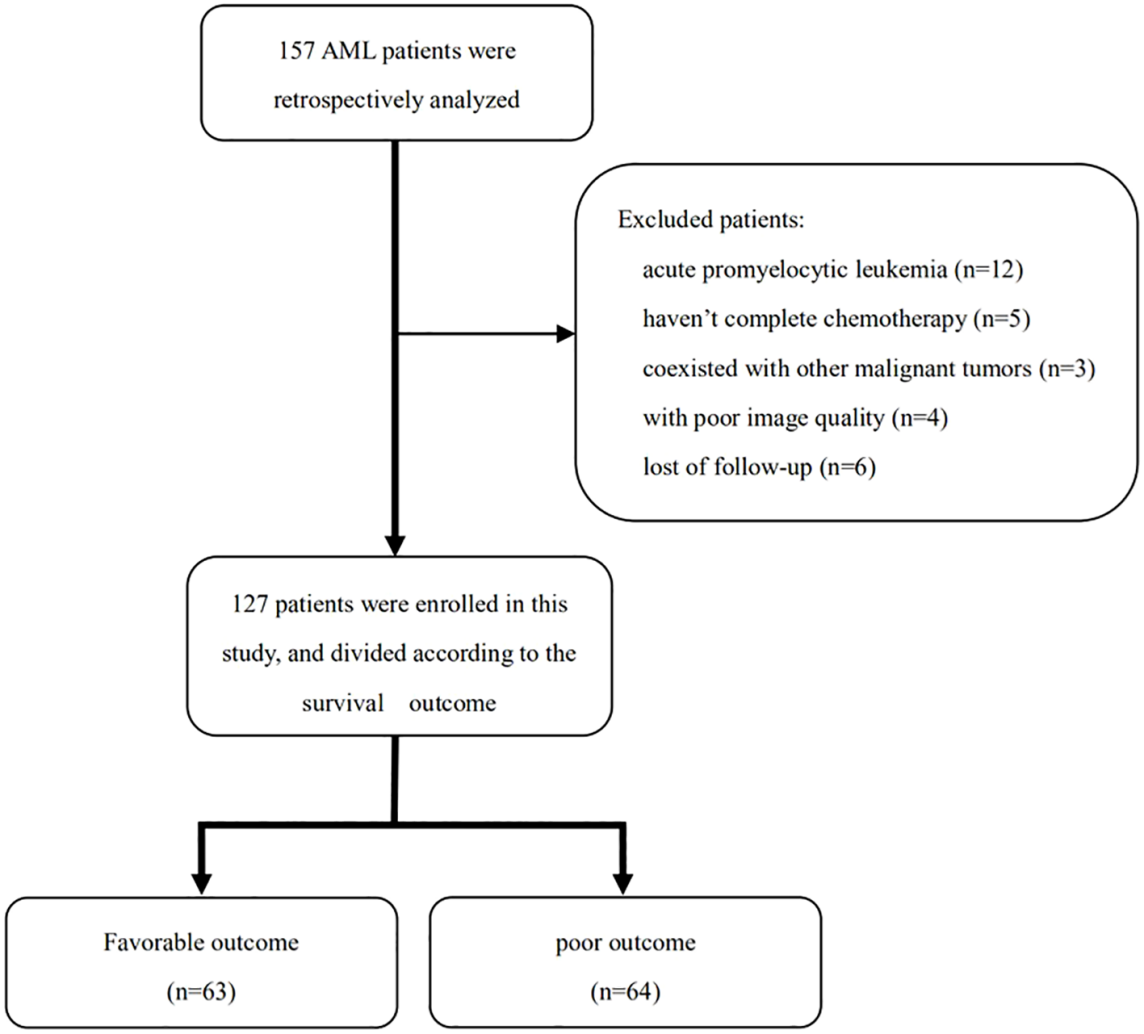
Flow diagram showing the inclusion of patients in the study.

The inclusion criteria of participants were as follows: (1) aged ≥ 18 years old; (2) AML diagnosed by bone marrow aspiration at the first visit (2); and (3) received the entire cycle chemotherapy schedule. The exclusion criteria were as follows: (1) diagnosis of acute promyelocytic leukemia; (2) incomplete chemotherapy; (3) coexistence with other malignant tumors; (4) coexistence with the following conditions: severe musculoskeletal disorders such as multiple vertebral fractures and multiple myeloma, long-term use of drugs that affect bone and system metabolism, and severe cardiopulmonary disease leading to decreased motor function; (5) poor image quality; and (6) lost to follow-up (**Figure 1**).

### Prognosis evaluation

The time from the date of complete remission to the date of AML relapse or death from any cause was defined as disease-free survival (DFS). OS was defined as the time from the date of definitive diagnosis to the date of the last follow-up or death (2). The patients’ data, including age, sex, height, weight, white blood cell count, European LeukemiaNet (ELN) risk stratification, minimal residual disease (MRD), and treatment schedule, were collected from the hospital information system. Risk stratification was performed according to the 2022 guidelines (9). The follow-up term was ≥ 2 years. An OS shorter than the median value was defined as a poor survival prognosis, whereas an OS equal to or longer than the median value was defined as a favorable survival prognosis (10).

### QCT acquisition and imaging analysis

QCT examination was performed with a 64-slice multidetector-row scanner (uCT780, United Imaging, Shanghai, China), Mindways QCT-Pro 6.0, and a standard model 4 QCT phantom (Mindways Software Inc., Austin, TX, USA). Scanning parameters included the following: voltage, 120 kV; automatic tube current, 100 mA; slice thickness, 1.25 mm; slice interval, 1.25 mm; helical pitch, 1.0875; scanning field of view, 350 mm; matrix, 512 × 512. Postprocessing of QCT was performed on the QCT Pro workstation.

**Figure 2:**
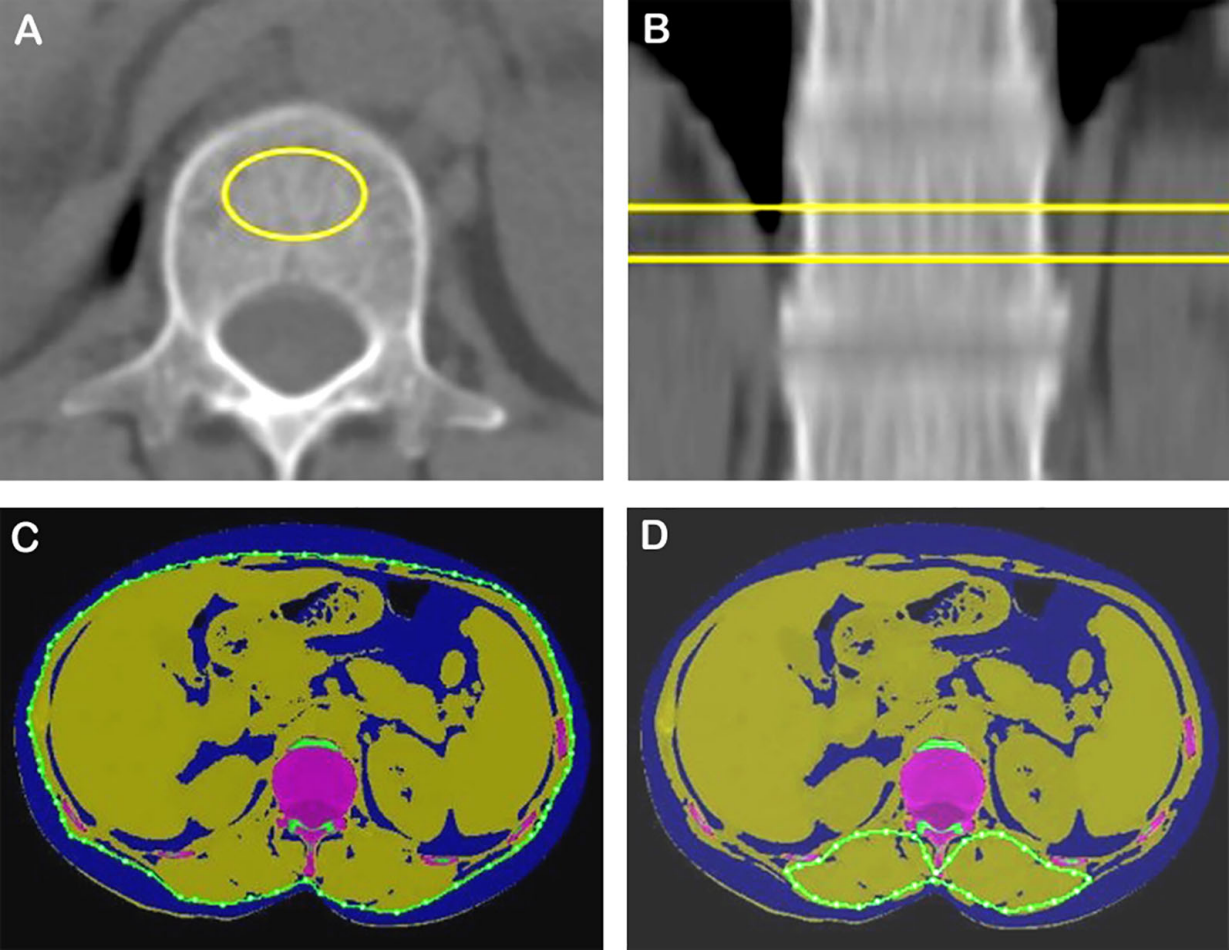
vBMD, skeletal muscle, and adipose tissue areas quantified using a single CT section at the first lumbar level. **(A, B)** Measurement of vBMD. **(C)** Assessment of SAT and VAT (blue). **(D)** Measurement of posterior vertebral muscle. vBMD, volume bone mineral density.

All QCT data were obtained from a conventional chest CT scan before treatment. The following QCT metrics were measured on axial images at the first lumbar vertebral body: volumetric bone mineral density (vBMD), area of para-vertebral muscles, area of subcutaneous adipose tissue (SAT), and area of visceral adipose tissue (VAT) (**Figure 2**). The skeletal muscle index (SMI), subcutaneous adipose index (SAI), and visceral adipose index (VAI) were calculated as the area of para-vertebral muscles, SAT, and VAT divided by height squared (m^2^), respectively (3). Osteopenia was defined as vBMD of 80–120 mg/cm^3^, and osteoporosis was defined when vBMD was less than 80 mg/cm^3^ (11). The attenuation of skeletal muscle ranges from − 29 to 150 HU, and adipose tissue ranges from − 190 to − 30 HU (12). The total adipose tissue (TAT) equals SAT plus VAT.

### Statistical analysis and development of a prediction model

All data were analyzed by using SPSS 26.0 (IBM, Chicago, IL, USA). Continuous variables were compared using Student’s *t*-test when the distribution was normal; otherwise, compared using the Mann–Whitney *U*-test. Categorical variables were compared using the Chi-square test. Receiver operating characteristic (ROC) analysis was applied to assess the cutoff value of SMI associated with death. Potential prognostic factors were considered in a Cox proportional hazards regression model in univariate and multivariate analyses. R software (version 4.2.2; http://www.Rproject.org) and MSTATA software (http://www.mstata.com/) were used to generate a nomogram. The ROC curve was used to analyze the prognostic predictive value of the nomogram. A total of 30% of patients were randomly allocated into a validation cohort for assessing the predictive performance of the nomogram. Calibration curves and decision curve analysis (DCA) were employed to evaluate the fitting degree and the net benefit under different thresholds. Inter-reader variability between radiologists was analyzed with the intraclass correlation coefficient (ICC). ICC > 0.75 was considered good consistency. A probability value of *p* < 0.05 was considered indicative of statistical significance.

## Results

### Patients’ characteristics

In the 157 AML patients initially screened, 30 patients were excluded for the following reasons: acute promyelocytic leukemia (*n* = 12), not having received standard treatment (*n* = 5), poor image quality (*n* = 4), coexistence with other malignant tumors (*n* = 3), and lost follow-up (*n* = 6) (**Figure 1**). Finally, 127 patients were retrospectively enrolled in this study, including 70 men (55.1%) and 57 women (44.9%), with an average age of 52.2 ± 15.3 years. The white blood cell count of these patients was 12.2 × 10^9^/L (3.5, 31.2). The median DFS and OS were 372 and 534 days, respectively. The ELN risk stratification was as follows: low risk (*n* = 49), medium risk (*n* = 22), high risk (*n* = 46), and not assessed (*n* = 10). The MRD of the 127 AML patients includes the following: negative (*n* = 51), positive (*n* = 69), and not assessed (*n* = 7). The treatment strategies included intensive chemotherapy (*n* = 66), nonintensive chemotherapy (*n* = 50), and hematopoietic stem cell transplantation (*n* = 11) (**Table 1**).

**Table 1:** Baseline characteristics of AML patients according to different prognostic groups (*n* = 127).

Variables	Favorable prognosis (*n* = 63)	Poor prognosis (*n* = 64)	*t*/*χ* ^2^/*Z* values	*p*-values
Age (years)	46.1 ± 13.5	55.6 ± 15.3	− 2.243	0.027
Sex (male)	39/63 (61.9%)	31/64 (48.4%)	2.328	0.127
Initial WBC	8.1 (3.3, 27.6)	13.0 (4.7, 45.0)	− 0.998	0.318
BMI				
BMI < 20	4/63 (6.3%)	7/11 (10.9%)	1.002	0.606
20–24.9	32/63 (50.8%)	33/65 (51.6%)		
≥25	27/63 (42.9%)	24/51 (37.5%)		
Risk stratification^a^				
Low	33/60 (55.0%)	16/57 (28.1%)	10.089	0.006
Medium	11/60 (18.3%)	11/57 (19.3%)		
High	16/60 (26.7%)	30/57 (52.6%)		
Not assessed	3	7		
MRD				
Negative	33/62 (53.2%)	18/58 (31.0%)	6.039	0.014
Positive	29/62 (46.8%)	40/58 (69.0%)		
Not assessed	1	6		
Treatment strategies				
Intensive chemotherapy	37/63 (58.7%)	29/64 (45.3%)	4.660	0.097
Nonintensive chemotherapy	19/63 (30.2%)	31/64 (48.4%)		
Hematopoietic stem cell transplant	7/63 (11.1%)	4/64 (6.3%)		
SMI	13.7 ± 2.9	12.7 ± 2.8	2.022	0.045
VAI	48.6 ± 25.9	47.8 ± 24.6	0.182	0.856
SAI	29.3 (20.2, 48.9)	30.3 (17.7, 44.5)	− 0.241	0.681
vBMD				
vBMD > 120	57 (90.5%)	49 (76.6%)	4.453	0.035
vBMD ≤ 120	6 (9.5%)	15 (23.4%)		
TAT	238.9 ± 115.2	226.9 ± 127.9	0.555	0.580
VAT/TAT	62.4 (45.0, 68.3)	60.4 (47.3, 67.8)	− 0.123	0.902
Sarcopenia	43 (68.3%)	57 (89.1%)	8.212	0.004

*WBC*, white blood count; *BMI*, body mass index; *MRD*, minimal residual disease; *SMI*, skeletal muscle index; *VAI*, visceral adipose tissue; *SAI*, subcutaneous adipose index; *vBMD*, volume bone mineral density; *TAT*, total adipose tissue; *VAT*, visceral adipose tissue.

a Based on the guidelines of the European Leukemia Network.

QCT metrics of 127 AML patients were as follows: SMI, 13.2 ± 2.9; SAI, 30.0 (19.3, 46.3); VAI, 48.2 ± 25.2; TAT, 232.8 ± 121.4; and VAT/TAT, 60.7 (46.9, 68.1). The BMIs of these patients include the following: BMI < 20 (*n* = 11), 20 ≤ BMI < 25 (*n* = 65), and BMI ≥ 25 (*n* = 51).

### Comparison between different prognosis groups

Compared to the patients in the favorable prognosis group, those in the poor prognosis group were older (46.1 ± 13.5 vs. 55.6 ± 15.3, *p* = 0.027), had higher ELN risk stratification (26.7% vs. 52.6%, *p* = 0.006), higher positive rate (46.8% vs. 69.0%, *p* = 0.014). However, there was no significant difference for gender (*p* = 0.127), white blood cell count (*p* = 0.318), BMI (*p* = 0.606), and treatment strategies (*p* = 0.097) between the two groups (**Table 1**).

### QCT metrics

Compared to the patients in the favorable prognosis group, those in the poor prognosis group had lower SMI (13.7 ± 2.9 vs. 12.7 ± 2.8, *p* = 0.045) and a higher rate of vBMD ≤ 120 (9.5% vs. 23.4%, *p* = 0.035). However, there was no significant difference in VAI (*p* = 0.856), SAI (*p* = 0.681), TAT (*p* = 0.580), or VAT/TAT (*p* = 0.902) between the two groups (**Table 1**). Based on ROC analysis, the cutoff value of SMI was 15.74cm^2^/m^2^.

**Figure 3:**
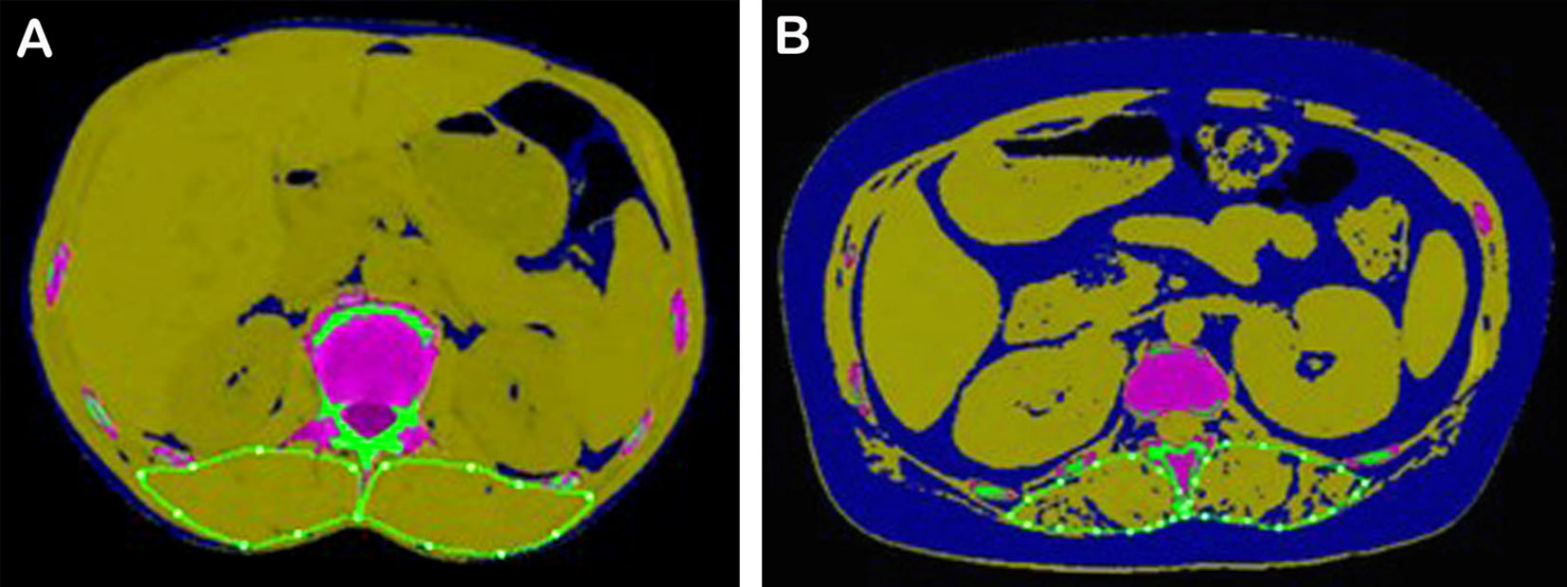
Typical AML patients with different survival prognoses. **(A)** A 54-year-old male patient with AML in the favorable prognosis group. His SMI was 16.95, ELN2022 was favorable, MRD was negative, OS was 1,002 days, DFS was 939 days, and his 2-year survival probability was 0.78. **(B)** A 40-year-old female patient with AML in the poor prognosis group. Her SMI was 9.97, ELN2022 was adverse, MRD was positive, OS was 384 days, DFS was 349 days, and her 2-year survival probability was 0.28. SMI, skeletal muscle index; ELN, European LeukemiaNet; MRD, minimal residual disease; OS, overall survival; DFS, disease-free survival.

The classic examples of AML cases with favorable and poor prognosis are shown in **Figure 3**.

### Prognostic factors

Univariate variable analyses showed that older age (*p <* 0.001), higher ELN risk stratification (*p* = 0.001), positive MRD (*p* = 0.004), SML < 15.74cm^2^/m^2^ (*p* = 0.011), and osteopenia or osteoporosis (*p* = 0.004) were significant factors of shorter OS. Similarly, older age (*p* = 0.001), higher ELN risk stratification (*p <* 0.001), positive MRD (*p* = 0.001), lower SMI (*p* = 0.012), and osteopenia or osteoporosis (*p* = 0.014) were significant factors of shorter DFS (**Tables 2**, **3**).

**Table 2:** Univariate and multivariate analyses of OS in AML patients before chemotherapy (*n* = 127).

Characteristics	Univariate analysis		Multivariate analysis	
	HR (95% CI)	*p*-value	HR (95% CI)	*p*-value
Age	1.028 (1.013–1.044)	< 0.001^*^	1.022 (1.005–1.039)	0.013
MRD	2.047 (1.263–3.318)	0.004^*^	2.826 (1.613–4.953)	< 0.001
Risk stratification^a^	2.379 (1.437–3.940)	0.001^*^	2.750 (1.584–4.772)	< 0.001
SMI	0.902 (0.834–0.977)	0.011^*^	0.893 (0.820–0.972)	0.009
vBMD	2.153 (1.272–3.643)	0.004^*^	1.342 (0.712–2.527)	0.363

*OS*, overall survival; *MRD*, minimal residual disease; *SMI*, skeletal muscle index; *vBMD*, volume bone mineral density.

a Based on the guidelines of the European Leukemia Network.

*p<0.05.

**Table 3:** Univariate and multivariate analyses of DFS in AML patients before chemotherapy (*n* = 127).

Characteristics	Univariate analysis		Multivariate analysis	
	HR (95% CI)	*p*-value	HR (95% CI)	*p*-value
Age	1.026 (1.011–1.041)	0.001^*^	1.017 (1.001–1.033)	0.035
MRD	2.142 (1.365–3.362)	0.001^*^	2.954 (1.768–4.933)	< 0.001
Risk stratification^a^	2.392 (1.504–3.802)	< 0.001^*^	2.745 (1.663–4.532)	< 0.001
SMI	0.910 (0.845–0.980)	0.012^*^	0.905 (0.838–0.977)	0.010
vBMD	1.915 (1.140–3.215)	0.014^*^	1.335 (0.731–2.439)	0.348

*OS*, overall survival; *MRD*, minimal residual disease; *SMI*, skeletal muscle index; *vBMD*, volume bone mineral density.

a Based on the guidelines of the European Leukemia Network.

### Multivariate variable analysis

**Figure 4:**
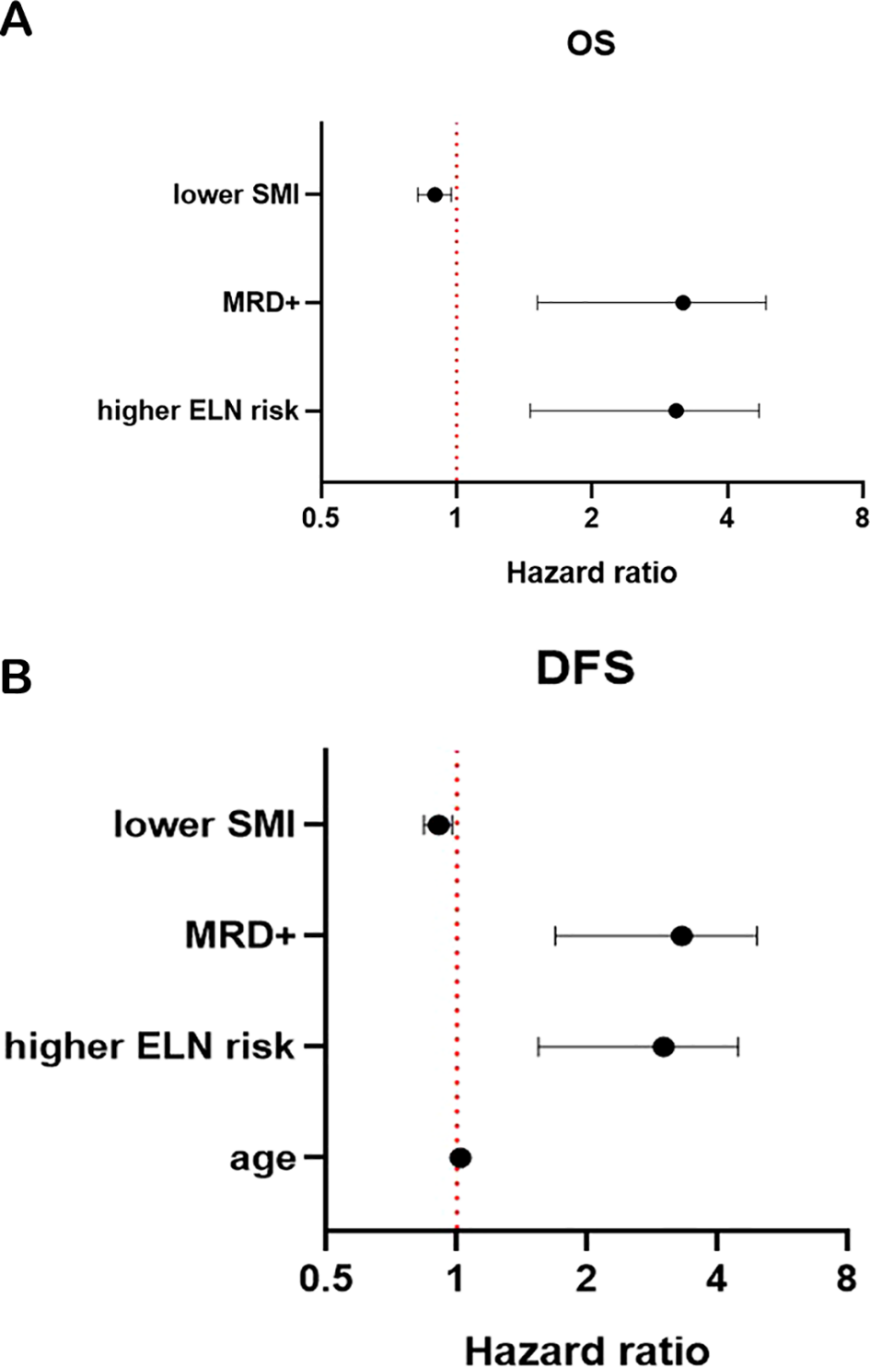
Forest plot for OS **(A)** and DFS **(B)**. SMI, skeletal muscle index; MRD, minimal residual disease; OS, overall survival; DFS, disease-free survival.

Higher ELN risk stratification (*p* < 0.001), positive MRD (*p* = 0.004), and SML < 15.74 cm^2^/m^2^ (*p* = 0.010) were significant factors of shorter OS. Older age (*p* = 0.018), higher ELN risk stratification (*p <* 0.001), positive MRD (*p* = 0.001), and lower SMI (*p* = 0.011) were significant factors of shorter DFS (**Tables 2**, **3**; **Figures 4A, B**).

### Prognostic utility of various models and nomograms

The variables that showed significance in multivariate analysis were included in the following survival prognosis prediction models: clinical model (age, MRD, ELN risk stratification), imaging model, and combination model (clinical and imaging variables). The areas under the ROC curve (AUCs) of the clinical, imaging, and combination models for predicting shorter OS in AML patients were 0.773, 0.627, and 0.797, respectively. The sensitivity, specificity, and Youden index of the combination model for predicting shorter OS were 0.791, 0.682, and 0.473, respectively. The AUCs of the clinical, imaging, and combination models for predicting shorter DFS in AML patients were 0.776, 0.628, and 0.798, respectively. The sensitivity, specificity, and Youden index of the combination model for predicting shorter DFS were 0.821, 0.697, and 0.517, respectively.

**Figure 5:**
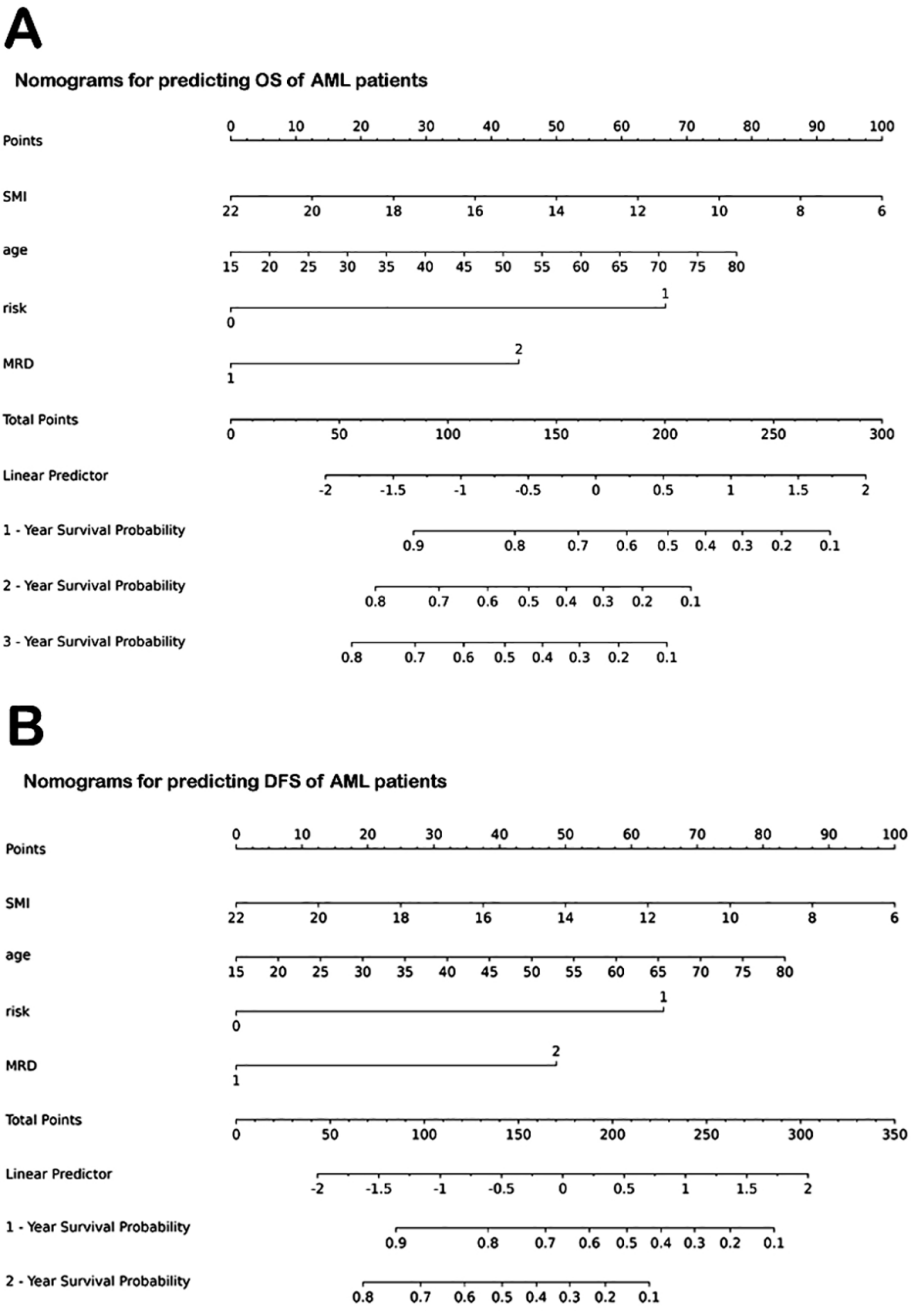
Nomograms for predicting OS and DFS of AML patients. **(A)** Nomogram for predicting OS. **(B)** Nomogram for predicting DFS. OS, overall survival; AML, acute myeloid leukemia; SMI, skeletal muscle index; MRD, minimal residual disease. DFS, disease-free survival.

**Figure 6:**
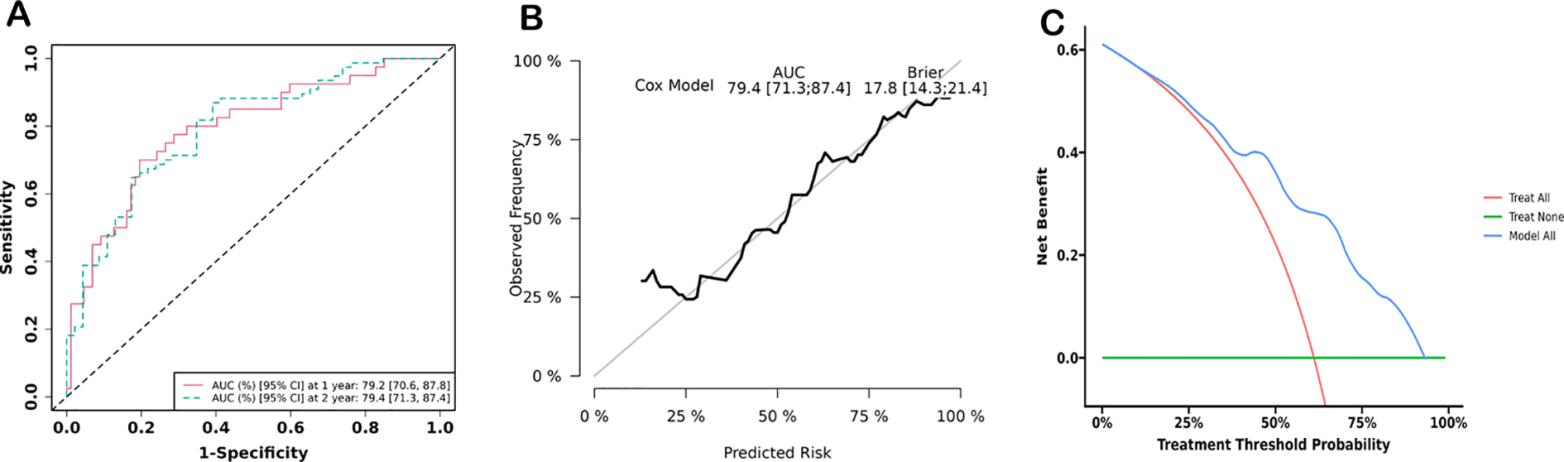
Prediction efficiency analyses. **(A)** Receiver operator curve (ROC) of the nomogram for predicting 1- and 2-year survival of AML patients. **(B)** Calibration curves of nomogram models for predicting 2-year survival. **(C)** Decision curve analysis of nomogram models for predicting 2-year survival.

The significant variables from multivariate analysis, including lower SMI, older age, and higher ELN risk stratification, were included to construct a nomogram for predicting OS and DFS (**Figures 5A, B**). The AUCs of the nomogram for predicting OS at 1 and 2 years were 0.792 (95% confidence interval [CI]: 0.706, 0.878) and 0.794 (95% CI: 0.713, 0.874), respectively (**Figure 6A**). The calibration curve showed a high coincidence and good fit between the nomogram model and the ideal curve (**Figure 6B**). DCA demonstrated that the nomogram model provided a significant net benefit in predicting survival prognosis in AML patients (**Figure 6C**).

### Consistency test and validation

The ICCs between the two radiologists for evaluating various QCT metrics were as follows: vBMD, 0.977 (95% CI: 0.967–0.984, *p* < 0.001); area of para-vertebral muscles, 0.973 (95% CI: 0.961–0.981, *p* < 0.001); SAT, 0.994 (95% CI: 0.992–0.996, *p* < 0.001); VAT, 0.986 (95% CI: 0.980-0.990, *p* < 0.001).

Thirty percent of patients (*n* = 38), including 18 patients from the favorable prognosis group and 20 patients from the poor prognosis group, were randomly selected to validate the performance of the three predicting models. The AUCs of clinical, imaging, and combination models for predicting shorter OS in the validation cohort were 0.778, 0.624, and 0.791, respectively. The sensitivity, specificity, and Youden index of the combination model for predicting shorter OS of the validation cohort were 0.611, 1.000, and 0.611, respectively.

## Discussion

In this study, we analyzed the association between QCT body composition metrics, clinical variables, and survival prognosis in patients with AML. A nomogram was constructed to individually predict the survival outcomes in AML. The study showed that sarcopenia diagnosed by QCT, along with clinical variables including higher ELN risk stratification and positive MRD, were significant factors for shorter OS. In addition, this study demonstrated the feasibility of using QCT metrics and clinical variables to develop a nomogram for predicting survival in AML patients, as DCA indicated a net benefit of the nomogram. Thus, these findings suggest that the nomogram, which combines pre-chemotherapy QCT metrics and clinical variables, is a potential tool to improve the prediction of survival prognosis in AML.

Our method has several advantages. First, the QCT data of AML patients were obtained through routine chest CT scanning in a one-stop mode. In clinical practice, chest CT is routinely performed for AML patients prior to treatment to assess underlying heart and lung conditions in many institutions. In this study, the QCT phantom was placed beneath the scanner mattress, allowing the data required for body composition analysis to be obtained simultaneously during chest CT scanning. Therefore, QCT body composition analysis can be used as a routine examination for AML patients without increasing radiation exposure or economic burden. Second, QCT quantitative metrics, including muscle area, adipose tissue and attenuation, and vBMD, are directly derived from high-resolution CT images (7). These metrics thus offer the advantages of accurate localization, objectivity, repeatability, and ease of comparison. Third, previous studies have shown that information extracted from cross-sectional images at the level of the first lumbar vertebra can reflect whole-body composition (8, 13, 14). In the present study, we segmented abdominal muscle, adipose tissue, and bone structures using QCT Pro software and confirmed the reliability of this method. Therefore, QCT metrics derived from conventional chest CT can be used as objective factors for predicting survival prognosis in AML patients.

It is not surprising that sarcopenia was identified as an independent risk factor for poor survival prognosis in AML in this study. We found that lower SMI was associated with both poor DFS and poor OS. Moreover, the incidence of sarcopenia was higher in the poor prognosis group (89.1%) than in the favorable group. The relationship between sarcopenia and prognosis in malignant tumors is a current focus of research. Previous studies have shown that sarcopenia is a significant predictor of poor prognosis in patients with malignant tumors following chemotherapy, surgery, and trauma (15, 16). Nakamura et al. (2) assessed the prognosis of 90 AML patients before treatment and found that the total area of abdominal muscles at the third lumbar vertebra on QCT was associated with shorter DFS and OS. In another QCT study involving 96 AML patients, Jung et al. (3) reported that patients with sarcopenia had shorter OS and higher mortality. The mechanisms by which sarcopenia contributes to poor survival prognosis in AML include increased catabolism of muscle proteins due to tumor cell proliferation, leading to elevated resting energy expenditure and a hypermetabolic state (17, 18). On the other hand, intensive chemotherapy often induces gastrointestinal adverse reactions, which can further aggravate nutritional deficiencies. Therefore, AML patients with sarcopenia often have a poorer survival prognosis. In addition, previous studies have shown that sarcopenia can be preceded by mild body composition abnormalities, referred to as “pre-sarcopenia” in the early stages of the disease (19). Therefore, the future research significance of QCT muscle metrics may lie in the early detection of muscle mass decline, thereby providing an early warning for sarcopenia.

Interestingly, we did not find a predictive value for adipose tissue-related metrics in the survival prognosis of AML. Although lower VAI and higher SAI were observed in the poor prognosis group, the differences in these adipose tissue metrics between the poor and favorable prognosis groups were not statistically significant. Similarly, Nakamura et al. (2) did not observe a significant decrease in adipose tissue in AML patients with shorter OS (*n* = 90) and concluded that adipose tissue-related metrics were not an independent factor of OS, DFS, and event-free survival (EFS). However, Nakamura et al. (3) reported that lower VAI and SAI were independent predictors of shorter OS and DFS in 96 AML patients. This discrepancy may be due to the fact that both the present study and Nakamura’s study included first-visit patients, whereas 14.6% of patients in Jung’s study (3) had secondary AML. It can be inferred that, in high-risk stratification patients, significant changes in adipose tissue metrics may impact the survival prognosis of secondary AML patients. Contrary to the abovementioned studies focusing on adipose tissue loss, other studies have shown that increased adipose tissue is also associated with poorer survival prognosis in hematologic malignancies. In a study of 129 patients with diffuse large B-cell lymphoma, Shen et al. (20) found that a larger area of VAT was associated with shorter OS. The mechanism by which increased adipose tissue contributes to poor survival prognosis may involve adipocytes impairing the efficacy of chemotherapeutic agents and promoting chemotherapy resistance (21). Overall, views regarding the relationship between adipose tissue content and survival prognosis in AML vary considerably. The main reasons for this divergence may include a nonlinear relationship between adipose tissue content and survival prognosis, as well as the lack of an accepted cutoff value for distinguishing between adipose tissue decrease and increase.

To the authors’ knowledge, this study is the first to explore the association between vBMD and the prognosis of AML. The present study showed a higher incidence of osteopenia/osteoporosis in the poor prognosis group. However, vBMD has not yet been proven to be an independent predictor of poor survival in AML patients. Bone marrow density (BMD) has previously been compared between AML patients and normal controls. Kumasaka et al. (22) assessed 56 AML patients and found that higher bone marrow CT attenuation values in AML patients compared with controls suggested bone marrow infiltration by leukemia cells replacing normal fatty marrow. vBMD was more sensitive than CT attenuation in detecting the changes in bone marrow components; therefore, vBMD may reflect leukemic cell infiltration in the bone marrow.

In addition, sarcopenia and osteoporosis often coexist and exacerbate each other, resulting in poorer survival prognosis for patients (23). Osteoporosis and sarcopenia may share several pathophysiological mechanisms, including myostatin, Wnt-β-catenin signal pathway regulation, deficiencies in certain nutrients such as vitamin D, and inflammatory cytokine activity (24). However, the present study did not confirm the association between SMI and vBMD in AML patients. This phenomenon may be due to the relatively small sample size. Therefore, the association between muscle metrics and bone mineral density, as well as their impact on survival prognosis, should be investigated in a large multicenter cohort in the future.

Moreover, aside from CT-derived QCT metrics, we also confirmed the prognostic value of other clinical variables, such as age, ELN risk stratification, and MRD. Older patients with AML would have a higher incidence of complications during treatment, which negatively impacts their survival prognosis. Our findings were generally consistent with those of prior studies (25). In the present study, the risk of death increased by 2.8% for each additional year of age. The prognostic value of MRD has previously been demonstrated, with positive MRD being associated with poorer prognosis (26). The present study also showed that positive MRD after chemotherapy was the most prominent predictor of shorter OS (HR = 2.826). Higher ELN risk stratification was associated with poorer prognosis. In this series, the risk of death increased by 175.0% in the middle-high-risk group compared with the low-risk group. Previous studies have also shown that patients with low ELN risk stratification have longer OS and DFS (27).

In this study, QCT body composition metrics were integrated with the clinicopathological variables of AML as a novel nomogram to evaluate the survival prognosis. Our nomogram model, which included QCT metrics, could improve prognosis prediction better than that of clinical variables alone, as evidenced by our AUC values. A nomogram is a novel tool for disease prediction and is essentially a visual representation of Cox regression. The total score of the nomogram is the sum of scores of all related variables, determined by their regression coefficients. Based on this total score, the probability of clinical prognosis for each patient can then be calculated (28). This study also confirmed the effectiveness of the nomogram in evaluating the survival prognosis of AML patients by combining QCT body composition metrics and clinical variables. The calibration curve of the nomogram model in predicting 1- and 2-year OS showed good fit, with minimal deviation between the predicted and actual results. These findings suggest that this nomogram has a potential value in predicting the prognosis of shorter OS in AML patients. As a concise and easily interpreted tool, a nomogram can be used to evaluate the patient’s condition, diagnose or predict disease risk or prognosis using multiple variables, and provide precise survival probabilities for patients with AML. Therefore, a nomogram could assist the physicians in developing individualized treatment strategies (29).

This study had several limitations. First, the quantitative evaluation of muscle by QCT could not assess muscle strength and function. However, the occurrence of sarcopenia precedes muscle strength and function loss (19). Therefore, QCT-derived muscle metrics may be helpful for the early detection of muscle abnormalities. Second, the lack of an accepted cutoff value for sarcopenia and adipose tissue loss results in poor comparability among different studies. Thus, the practicability of QCT body composition metrics is less favorable. Third, this was a single-center study with a small sample size, and the results need to be verified by studies with large multicenter samples in the future. However, we randomly selected certain patients for verification and found similar prediction efficacy compared with the results of the whole cohort.

## Conclusions

In summary, we presented a QCT-clinical nomogram utilizing chest CT images to predict prognosis in patients with AML. The combination of QCT metrics and clinical variables yielded a survival probability prediction system that could stratify AML patients. Our data suggest that information from the QCT-clinical nomogram could serve as a biomarker to assist clinicians in selecting optimal therapies for the personalized treatment of AML, although this observation requires further validation.

## Data Availability

The datasets presented in this article could be provided upon application. Requests to access the datasets should be directed to quanguanmin@hebmu.edu.cn.
